# Artificial intelligence enabled smart mask for speech recognition for future hearing devices

**DOI:** 10.1038/s41598-024-81904-y

**Published:** 2024-12-03

**Authors:** Hira Hameed, Lubna, Muhammad Usman, Jalil Ur Rehman Kazim, Khaled Assaleh, Kamran Arshad, Amir Hussain, Muhammad Imran, Qammer H. Abbasi

**Affiliations:** 1 James Watt School of Engineering, University of Glasgow, Glasgow, G12 8QQ UK; 2 University of Engineering & Technology, UETP, Peshawar, Pakistan; 3 School of Computing, Engineering and Built Environment, Glasgow Caledonian University, Glasgow, G4 0BA UK; 4 Department of Electrical and Computer Engineering, College of Engineering and Information Technology, Ajman University, Ajman, UAE; 5 School of Computing, Edinburgh Napier University, Edinburgh, Scotland, UK; 6 Artificial Intelligence Research Center (AIRC), Ajman University, Ajman, UAE

**Keywords:** Health care, Engineering

## Abstract

In recent years, Lip-reading has emerged as a significant research challenge. The aim is to recognise speech by analysing Lip movements. The majority of Lip-reading technologies are based on cameras and wearable devices. However, these technologies have well-known occlusion and ambient lighting limitations, privacy concerns as well as wearable device discomfort for subjects and disturb their daily routines. Furthermore, in the era of coronavirus (COVID-19), where face masks are the norm, vision-based and wearable-based technologies for hearing aids are ineffective. To address the fundamental limitations of camera-based and wearable-based systems, this paper proposes a Radio Frequency Identification (RFID)-based smart mask for a Lip-reading framework capable of reading Lips under face masks, enabling effective speech recognition and fostering conversational accessibility for individuals with hearing impairment. The system uses RFID technology to make Radio Frequency (RF) sensing-based Lip-reading possible. A smart RFID face mask is used to collect a dataset containing three different classes of vowels (A, E, I, O, U), Consonants (F, G, M, S), and words (Fish, Goat, Meal, Moon, Snake). The collected data are fed into well-known machine-learning models for classification. A high classification accuracy is achieved by individual classes and combined datasets. On the RFID combined dataset, the Random Forest model achieves a high classification accuracy of 80%.

## Introduction

According to the World Health Organisation (WHO), normal hearing is the ability to detect sounds at a level of 20 decibels (dB). In contrast, the inability to hear sounds above 20 dB can be perceived as hearing loss. Hearing loss can range from mild to severe and when it becomes severe, the patient is classified as “deaf”. This can have a significant impact on communication and learning abilities. Hearing loss is thought to affect 430 million individuals worldwide or around 5% of the entire population. It is predicted that the number will rise to 700 million by the year 2050^1^. Around 11 million people in the UK have hearing impairments, with age-related hearing loss being a major concern^2^.

By 2050, next-generation hearing aids will need transformation that is unrestricted by speech or sound enhancement constraints. We humans require visual information in addition to sound to comprehend spoken words. Speech recognition depends on Audio and visual information, such as Lip-reading. In the domain of audio-video speech enhancement (AVSE), efforts are directed toward augmenting speech quality through the utilisation of visual information gathered by a camera^3^. Concurrently, the field of visual speech recognition revolves around lip-reading using exclusively visual indicators without dependence on audio data^4^. Additionally, a pioneering framework^5^ for lip-reading using a mobile phone’s front camera is developed by employing convolutional neural networks (CNN) and temporal neural networks (TCN) to forecast Greek phrases. Unfortunately, the audio is susceptible to issues in noisy environments, making it challenging to recognise a person’s voice. The integration of cameras in hearing aids for collecting visual information raises privacy concerns that could prevent their widespread use. These devices may be seen as filming people without their consent, which is illegal in many parts of the world. Additionally, face masks have limited the effectiveness of vision-based hearing aids, especially in the age of COVID-19. Hence, a viable solution is to develop a contactless Radio Frequency (RF) sensing method to detect Lip and mouth movements. This will offer highly accurate cues to hearing aids by distinguishing spoken sounds and recognising speech patterns using machine learning algorithms. One of the key advantages of RF sensing-based lip-reading over vision-based systems is its ability to operate effectively even in situations where face masks are present, and it can detect visual cues such as Lip and mouth movements. By incorporating a single antenna into the design of a hearing aid and reader to receive data from the RFID tag, RF sensing offers an exciting opportunity to transform next-generation hearing aids into multi-modal devices. The authors of this paper have created a solution and tested the operation of a smart face mask that uses RFID technology to detect spoken sounds.

With variations in amplitudes of RSSI (Received Signal Strength Indicator) caused by Lip and mouth movements, signal patterns corresponding to spoken sounds can be easily mapped. These patterns can be classified into speech, words, phonemes, or spoken letters using ML algorithms. The RFID-based system uses RSSI values to classify different Lip movements. Lip-reading frameworks have a wide range of potential applications, including hearing aids, biometrics, and voice-enabled control systems for smart homes and automobiles.

Feature improvement techniques are examined to reduce speaker variability and compared low-level, image-based features and high-level, model-based features for Lip-reading are explored^6^. As researched^7^, the high-level Active Appearance Models (AAM) based features significantly outperform the low-level features. In this regard, different Lips images were captured from a standard camera while performing letter and performed different input features which were then fed to Neural Network for recognition. Explainable AI^8^ is reviewed within healthcare, underlining its capacity to boost clarity, foster trust, and augment decision-making processes in clinical care and medical investigations. The audio-visual databases are effectively utilised for Lip-reading exploring the applications of different deep-learning architectures for classification^9^. Studies showed that the Profile View (PV) Lip-reading had a significant advantage over the Frontal View (FV). Integration of audio and visual features resulted in improved speech recognition^10^. Researchers used two types of RF sensing, namely radar and Wi-Fi, to detect Lip movements and applied various machine learning and deep learning algorithms for the classification of spoken vowels^11,12^. A study^13^ aimed to provide a speech recognition technique using a portable auditory radar operating at 24 GHz and a webcam. Participants were asked to speak the English letter “A” only. Another research proposed^14^ a Lip-reading recognition system based on Channel State Information (CSI). In this system, mouth movements are processed in two stages. First, interference is filtered out, and then discrete wavelet packet decomposition is used to create mouth movement profiles. Machine learning techniques are used for pronunciation classification. In another related work^15^, Lip movements are decoded using flexible triboelectric sensors based on the structural principle and electrical properties. To make the sensors easily visible, they are positioned inside a pseudo mask that leaves the Lips uncovered. However, the recent RF system has some limitations, such as difficulty in localising the targeted subject when multiple subjects speak simultaneously.

A commercial RFID is utilised for speech recognition^16^ with multiple tags embedded on a transparent sheet to detect a single word. The system achieves an accuracy of 0.95% in detecting user speech and can recognise a vocabulary of 20 words with an average accuracy classification of 0.88%. Everyday objects’ sensing capabilities using long-range RFID in the IoT are identified in a study^17^, detecting user presence at 96.7% and daily activities at 82.8%. An RFID-based gesture recognition system is proposed^18^, achieving an experimental accuracy of 97.2% with 18 different gestures. Furthermore, RFID tattoos^14^ are also used for speech recognition. The proposed wafer-thin tattoos are attached around a user’s face and can be easily concealed with makeup. The RFID tag speech recognition system shown 86% accuracy with 10 users. However, people may feel discomfort wearing masks attached to the face and multiple tags may be required to record a single word, which can be expensive. Additionally, recording data from multiple users simultaneously can be challenging.

RFID has great use cases in speech applications, such as RF-Mic^19^ uses glasses equipped with an RFID tag to eavesdrop on speech by analyzing subtle facial movements. It processes RF signals, extracts speech dynamics through deep-learning models, and constructs a user-irrelevant eavesdropping model. Experiments demonstrate its effectiveness and accuracy in live voice eavesdropping. Moreover, an RFID-based assistive glove^20^ was developed to help visually impaired individuals identify objects and discern colors without tactile feedback. Being tested by 17 blindfolded participants, the glove achieved a 96.32% success rate, with 70% of users satisfied. Future improvements could include wireless headphones, waterproofing, and size reduction for broader applications. A very interesting article, “UltraSR: Silent Speech Reconstruction via Acoustic Sensing^21^” introduced UltraSR, a novel silent speech interface that reconstructed audible speech from silent articulatory gestures using ultrasonic signals on a portable smartphone. Addressing privacy and contact issues from previous SSI methods, UltraSR used multi-scale feature extraction, an end-to-end mapping model, cross-modal data augmentation, and user adaptation technique. It achieved a Character Error Rate as low as 5.22%. State-of-the-art applications in Wi-Fi, radar, SDR, and RFID-based sensing are also discussed in a survey^22^, including their advantages, limitations, and research gaps. This comprehensive study emphasise on the potential of contactless sensing for applications like independent assisted living and healthcare, emphasizing the need for further research in multi-subject detection and tracking, and smart world applications in the Internet of Things (IoT) domain, alongside contributions to the 5G and 6G industries and enhancements through machine learning.

There exists limited literature on RF sensing-based Lip movement detection, hence there is a need to develop a comprehensive dataset that includes a wide range of subjects, including diverse age and gender groups, and includes samples of vowels, consonants, and words. The aim of this study is to identify and differentiate between different lip readings using RSSI data obtained through an RFID tag. In the proposed work, fourteen types of RSSI data will be examined, including data relating to vowel sounds (A, E, I, O, U), consonants (F, G, M, S), and words (Fish, Goat, Meal, Moon, Snake). A Passive (UHF Textile Laundry) RFID tag is utilised for recording the dataset and stitched on a normal mask which is available in the common market. The embedded RFID tag inside the mask can be worn without hesitation, eliminating discomfort for subjects. The data gathered is represented in the form of RSSI values and various machine learning models, including Random Forest, K-Nearest Neighbors (k-NN), and Support Vector Machine (SVM) with Radial Basis Function (RBF) kernel are applied for classification purposes.

This work introduces a novel RFID-based washable smart mask designed for lip-reading recognition, which can automatically recognize and translate lip movements. The smart mask significantly improves speech recognition, enhancing conversational accessibility for individuals with hearing impairments. Various face masks, including single-use and surgical types with 1-ply, 2-ply, and 3-ply properties, as well as different colors, were employed for system authentication during data collection. The dataset comprises 2800 samples of 14 distinct types of lip-reading, captured at a distance of 0.50 meters. To facilitate analysis, the data was categorized into three sub-classes and collected from four participants, including two males and two females, aged between 16 and 50 years. In terms of performance, the RFID smart mask achieved an accuracy of 80.07% for vowels, 89.05% for consonants, 93.0% for word datasets, and 80% for all 14 classes using machine learning models.

The rest of the paper is divided into the following sections: Section “RFID tag performance setup and test results” discusses the testing approach of the proposed RFID tag. Section “Methodology” outlines the methodology adopted in this study, including details of the experimental setup, data collection and annotation, data pre-processing, machine learning algorithms, and the evaluation metrics for the classification model. Section “Results and discussion” discusses the results and discussion. Finally, Section “Conclusion and future works” concludes the paper and outlines future research directions.

## RFID tag performance setup and test results

The passive Ultra-High-Frequency (UHF) RFID tag used in our proposed smart mask underwent testing for reusability and rigor. It is a flexible, low-profile, linearly polarised textile laundry tag that offers versatile attachment methods and meets specific electrical specifications. The dimension of the tag is 58$\times$15$\times$1.5 mm. It is an EPC Gen2 compliance tag with a copper dipole antenna and Impinj Monza R6P Integrated Circuit (IC)/chip.

**Fig. 1:**
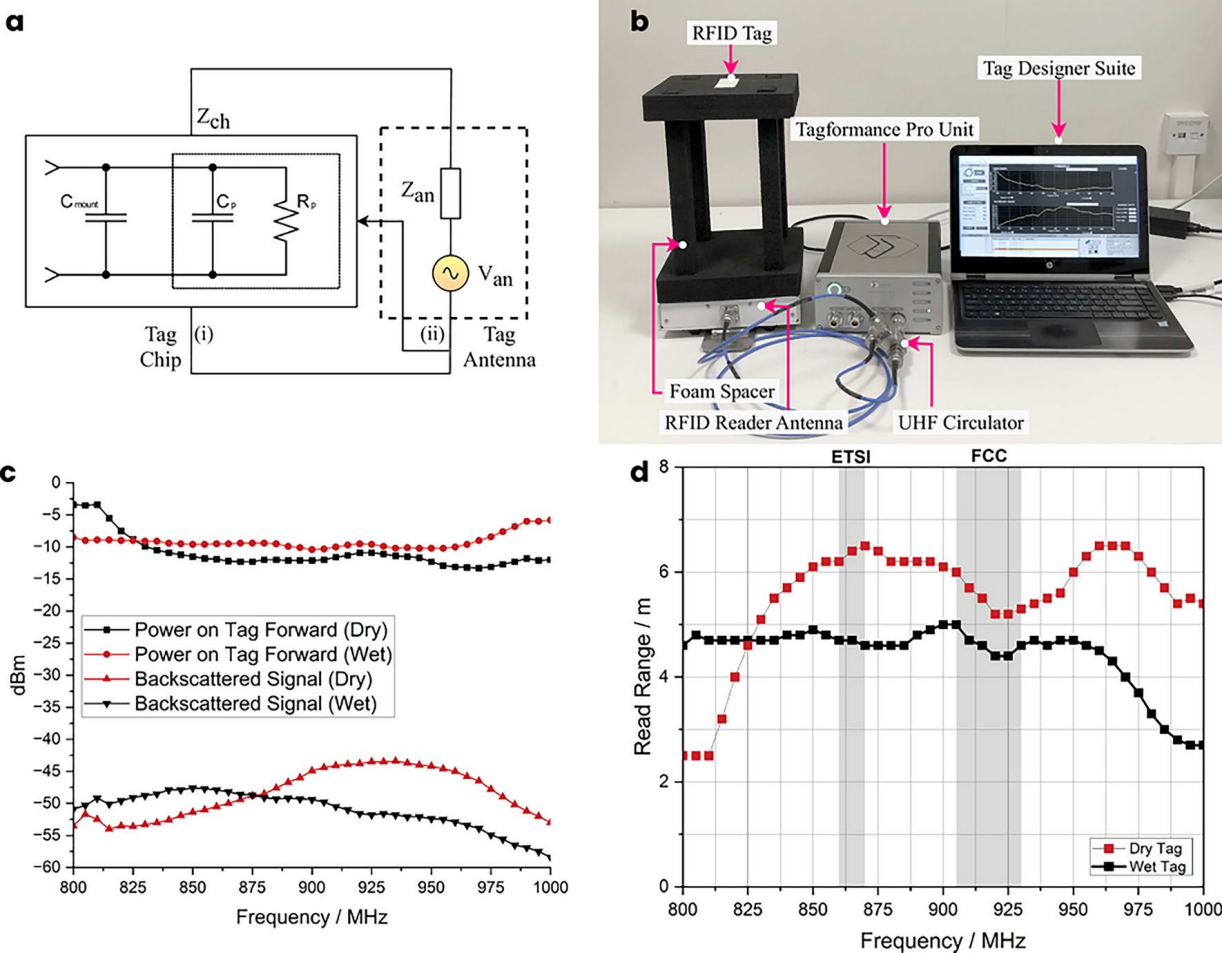
(**a**) Linearised RF-Model of the tag. (i) Tag chip lumped element model. (ii) Tag antenna lumped element model). (**b**) Experimental setup for tag measurements, using Tagformance Pro device. (**c**) Analysed power on tag forward, and backscatter signal at 800–1000 MHz with multiple transmit-power levels for both the dry and wet tag. (**d**) Read range measurements of the tag in both dry and wet conditions.

**Fig. 2:**
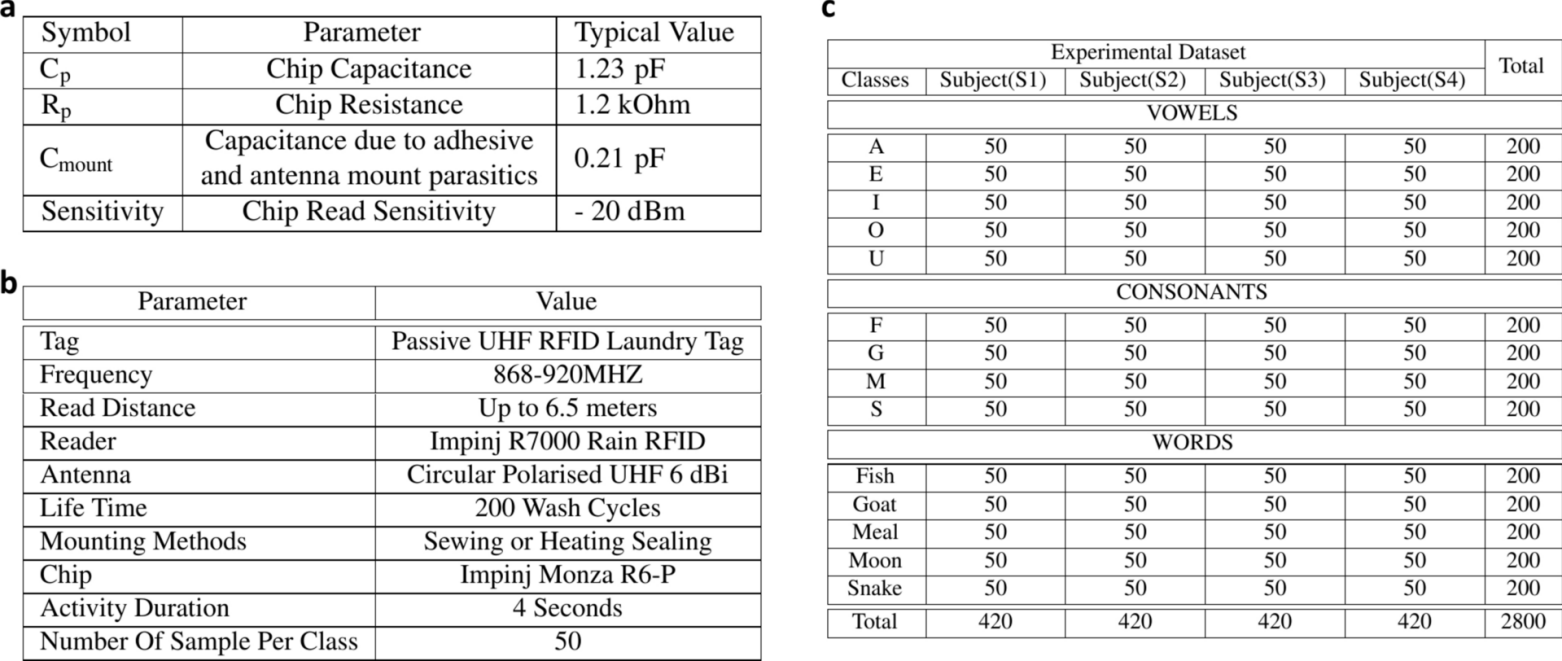
(**a**) Operating Conditions and Electrical Characteristics of Monza R6-P chip port model. (**b**) Selected hardware and software parameter settings. (**c**) An overview of the information gathered, the number of participants, and the activities performed.

A simplified model of the tag chip, consisting of lumped elements, is shown in Fig. 1a-(i). The port model is derived using a source-pull method due to the nonlinear and time-varying nature of the tag’s RF circuits. This model is an accurate mathematical representation of the chip’s behavior over a wide range of frequencies. Figure 2a, provides the values of the lumped elements for the Monza R6-P tag chip’s port model, which are valid for all primary regions of operation within the UHF range (868–920 MHz). The lumped elements include $C_{mount},$ which represents the parasitic capacitance resulting from the overlap of the antenna trace with the chip surface, $C_p,$ which is intrinsic to the chip and appears at the chip terminals, and $R_p,$ which represents the energy conversion and absorption of the RF circuits.

The chip impedance $Z_{ch}$ and antenna impedance $Z_{an},$ which vary with frequency, can be expressed according to^23–25^, and the equivalent lumped circuit depicted in Fig. 1a as:

1 $$\begin{aligned} Z_{ch}= & R_{ch} + jX_{ch} \end{aligned}$$

2 $$\begin{aligned} Z_{an}= & R_{an} + jX_{an} \end{aligned}$$

The chip and antenna resistance is represented by $R_{ch}$ & $R_{an},$ respectively, while the chip and antenna reactance is denoted by $X_{ch}$ & $X_{an}.$ *Vant* refers to the open-circuit RF voltage that arises from the electromagnetic field generated by the reader at the terminals of the tag antenna. The impedance of the chip, $Z_{ch}$, is affected by the power that the chip absorbs, $P_{ch}$, and this often has a draining effect on energy. To determine the power that is absorbed by the tag chip, $P_{ch}$, we utilise the maximum available power from the antenna, $P_{an}$, as well as the power transmission coefficient, $P_{ch}$, as shown below:

3 $$\begin{aligned} P_{ch} = P_{an}\tau \end{aligned}$$

The maximum antenna power, $P_{an}$, is achieved when $Z_{ch} = Z_{an}$. The power transmission coefficient, $\tau$, represents the degree of impedance matching between the IC and the antenna and is expressed as follows:

4 $$\begin{aligned} \tau = \frac{4*R_{ch}R_{an}}{Z_{ch}+Z_{an}} \end{aligned}$$

As $\tau$ approaches unity, the match between the tag chip and antenna impedance improves, with a perfect complex conjugate match achieved at $\tau$ = 1. Thus, for a given chip-and-tag antenna setup, an ideal situation would be where $Z_{ch}$ = $Z_{an}$, corresponding to $\tau$ = 1. Moreover, in order for the chip to activate, the antenna is often matched to the minimum threshold power, $P_{th}$.

The Friis free-space equation is utilised to compute the free-space tag antenna power, $P_{an}$, where:

5 $$\begin{aligned} P_{an} = P_{read}G_{ant}G_{read}\left( \frac{\lambda }{4\pi d}\right) ^2 \end{aligned}$$

Here, $P_{read}$ and $G_{read}$ refer to the reader-transmitted power and antenna gain, respectively. $G_{ant}$ represents the tag antenna gain, $\lambda$ denotes the wavelength, and *d* represents the distance between the tag and reader. Substituting Eq. (3) and determining the read range, *r*, at which the tag receives the minimum $P_{th}$ yields the following equation:

6 $$\begin{aligned} r = \frac{\lambda }{4\pi }\sqrt{\frac{P_{read}G_{ant}G_{read} \tau }{P_{th}}} \end{aligned}$$

The tag’s resonance, which represents the peak read range over a frequency range, is associated with the maximum power transmission coefficient, $\tau$. Therefore, in order to achieve the maximum read range, it is essential to optimise the tag antenna to achieve the highest power transmission coefficient, $\tau$, and then utilise (6) in conjunction with the reader system to calculate the corresponding read range, *r*. For a parallel circuit with a resistor and capacitor:

7 $$\begin{aligned} Q = R_p\times \omega \left( C_p + C_{mount}\right) \end{aligned}$$

From 2a, the parallel resistance of the tag chip, $R_p$ = 1200 $\Omega$, while the parallel capacitance, $C_p$ = 1.23 pF with an additional parasitic capacitance of 0.21 pF. Furthermore, $\omega =2 \pi f$, where *f* is the central frequency of 900 MHz. As per circuit theory, the real component of the chip impedance, $R_{ch}$, at this frequency can be calculated as $R_{ch} = {R_p}/({1+Q^2})$. For this particular case, $R_{ch}$ evaluates to 12.3 $\Omega$. Additionally, the imaginary component of the chip impedance is $1/(\omega Cp)$ and equals −120.8 $\Omega$. Utilising (1), we can express the chip impedance, $Z_{ch}$ = 12 − j121 $\Omega$. The matching of the antenna-chip impedance is validated through the read range tests, which are discussed in the subsequent section.

The chip employed in these tags is characterised by exceptional read sensitivity of up to −22.1 dBm when used with a dipole antenna. Additionally, the chip leverages autotune technology to maintain performance consistency across various dielectric materials. This technology’s primary advantage lies in its cost-effectiveness and high efficiency in achieving our research objectives. It has the capacity to store individualised data within the integrated IC and seamlessly integrates into IoT technologies. This functionality allows for the unique identification of items by leveraging the unique ID stored in its IC. However, a potential limitation of RFID technology is its restricted read range capability. The read range tests and other necessary measurements are presented in Section “RFID tag measurement results using tagformance^®^ pro unit”.

### RFID tag measurement results using tagformance^®^ pro unit

To evaluate the reliability of the RFID tag, we employed the Voyantic Tagformance^®^ Pro device used in the industry. The measurement setup is shown in Fig. 1b. The Tagformance device is composed of several components, including Tag Designer Suite (TDS)^1^ software, a Tagformance unit that comes with a UHF circulator and a foam spacer, and a linearly polarised RFID reader antenna that has a gain of 6 dBi and can be adjusted through the settings^26^.

In the read range test, the sensitivity of the RFID tag is assessed across a frequency range of 800–1000 MHz. At each frequency, the power of the forward and backscatter signals on the tag is analysed with various transmit-power levels. The test results are illustrated in Fig. 1c.

The read range measurements for the dry and wet tag are presented in Fig. 1d. The dry tag achieves a read range of up to 6.5 m. whereas the wet tag can be read at up to 5 m.

## Methodology

**Fig. 3:**
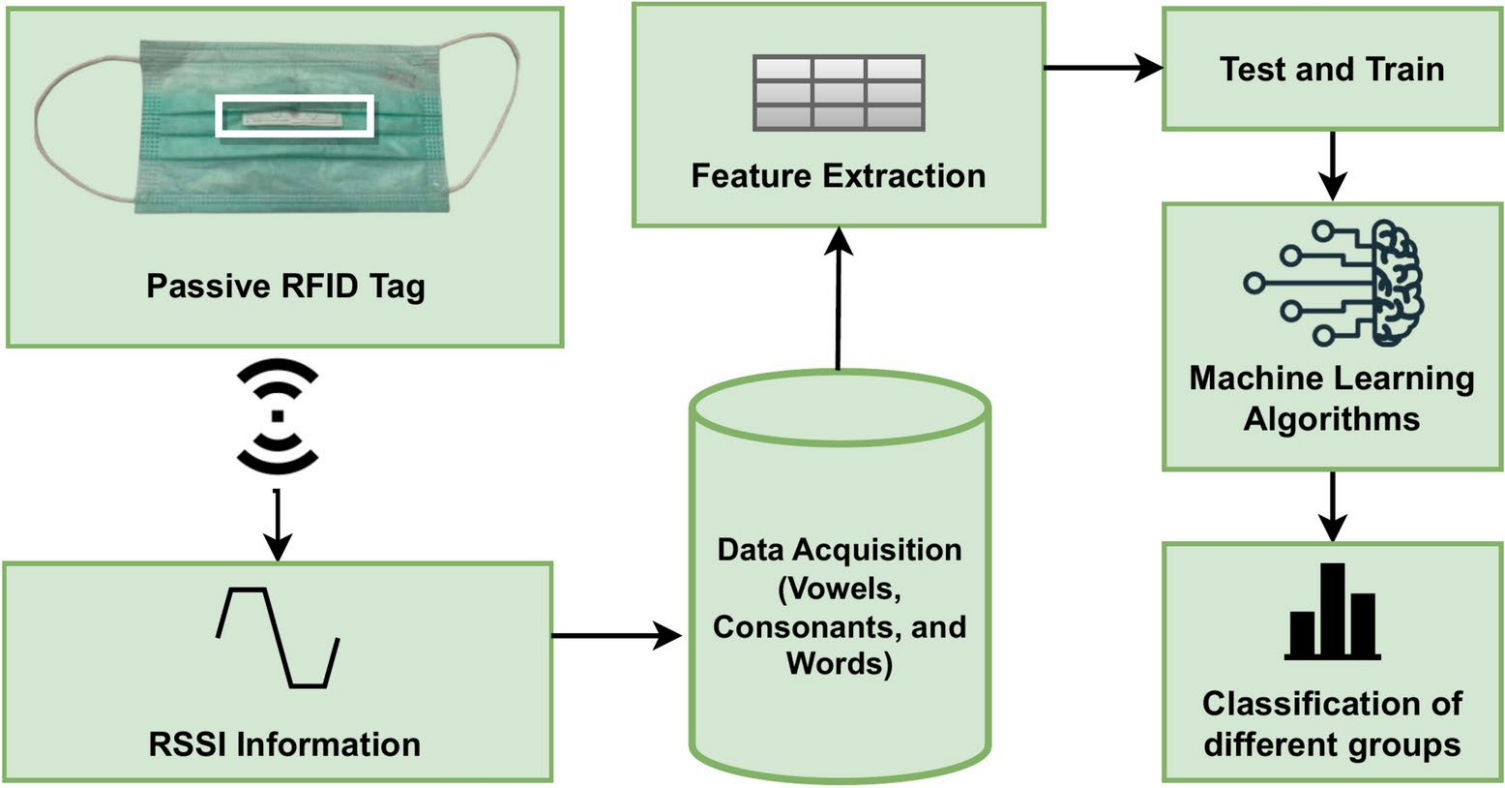
An methodoloy of the proposed framework signal flow diagram highlighting the RFID technology, data collection, and ML models for Lip-reading classification.

The block diagram in Fig. 3 shows the methodology used in this study. There are three steps to the suggested framework. In the first step, we collected, build and annotated various Lip-reading datasets. In the second step, the pre-processing phases are explained. Lastly, several machine learning models were used to classify the RFID-based Lip-reading. The following subsections provide a detailed description of each step of the proposed methodology.

**Fig. 4:**
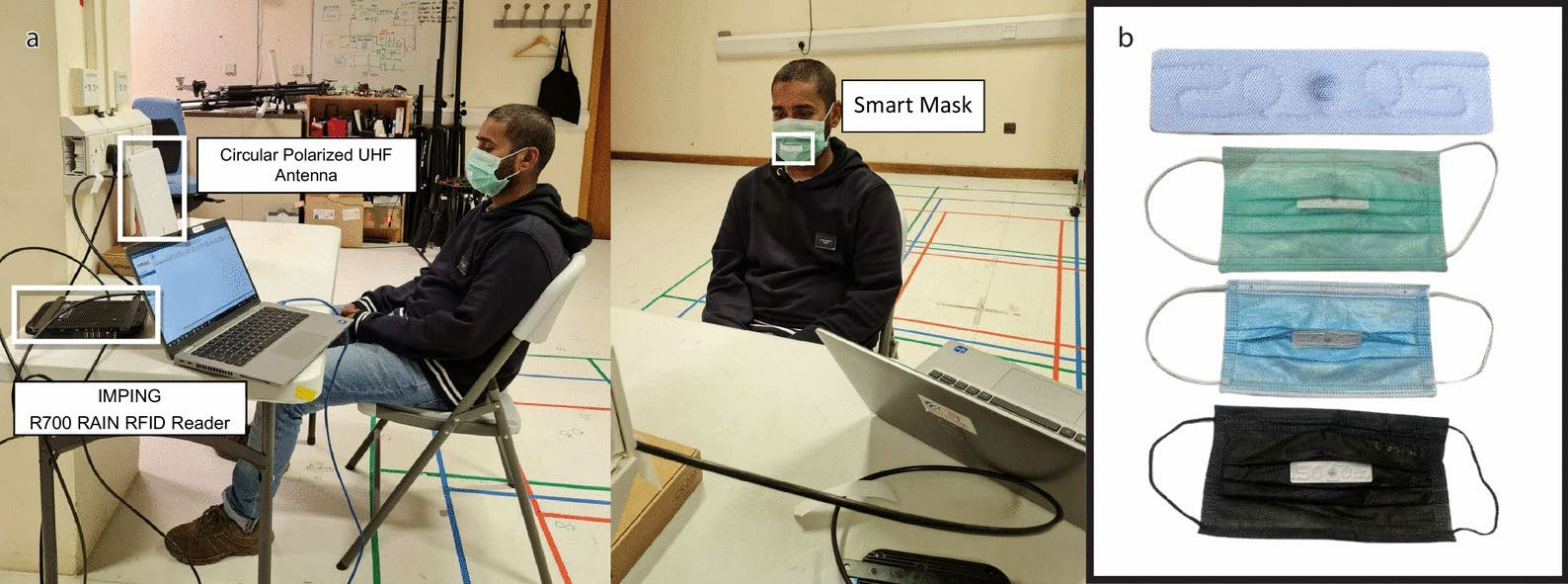
Experimental setup of Lip-reading data collection using RFID-based smart mask. (**a**) Real experimental setup. (**b**) Color-thickness variants of smart masks used in the experimental setup.

**Fig. 5:**
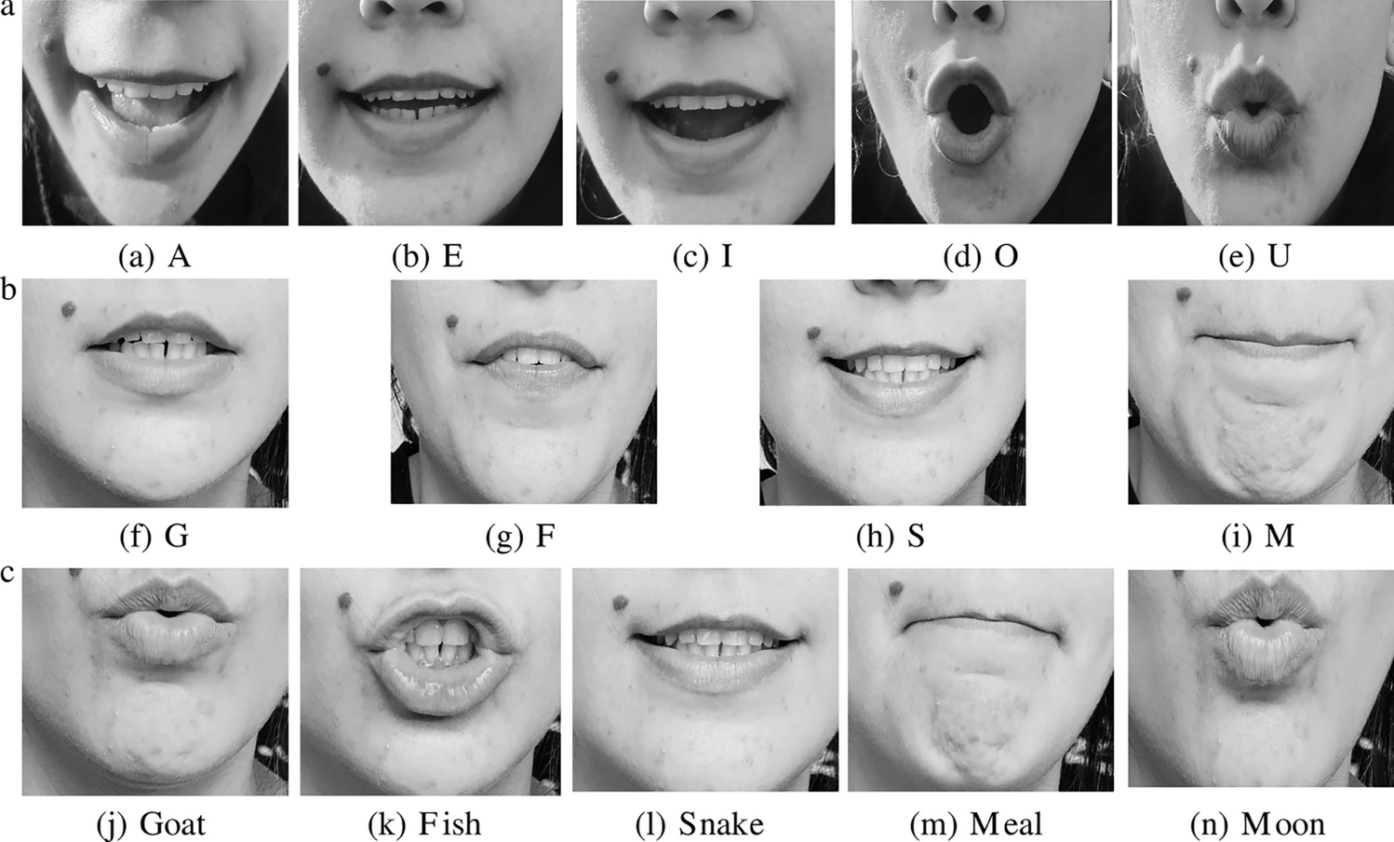
A visual illustration of the Lip-reading. (**a**) Vowels. (**b**) consonants. (**c**) Words.

**Fig. 6:**
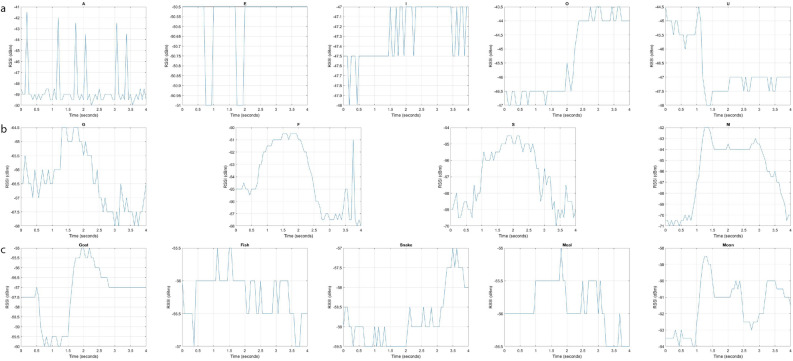
A graphical illustration of the received lip-reading signals: (**a**) vowels, (**b**) consonants, and (**c**) words.

### Experimental setup and data collection

In this step, we used an RFID-based smart mask to collect data on Lip-reading. The experimental setup of the Lip-reading using an RFID-based smart mask is shown in Fig. 4a. The RFID laundry tag was stitched on disposable face masks. The multiple color mask having different thicknesses were used for the experiments to check the authenticity of the system which is shown in Fig. 4b. The key parameter settings of the RFID Lip-reading system are indicated in Fig. 2b. In this system, participants were asked to sit 0.50 meters away from the RFID reader and antenna. The subject’s body was in its regular position during data collection, with only head movements. Furthermore, each activity had a time limit of 4 seconds and the data collection process involved recording a single word/vowel/consonant from each subject. Figures 5 and 6, provides a visual illustration of the pronounced vowels, consonants, and words^2^. A total of four participants, two males, and two females, participated in the data collection process. Multiple participants were invited to the data collection process to make the data more realistic and diverse. During the experiments, a total of 2800 data samples were collected, with 50 samples collected in each class. We distributed the dataset into three sub-classes (vowels, consonants, and words). Figure 2c provides a detailed overview of the collected dataset. In particular, each class is divided into two parts 80% data for training and 20% dataset for testing purposes. In each sub-set either vowels or words, a total of 1000 data samples were collected from participants, where 800 were utilised for training and 200 for testing purposes. In the case of consonants, a total of 800 data samples were collected from participants, where 640 were utilised for training and 160 for testing purposes.

### Dataset

A collection of RSSI values was produced as a result of the earlier described data collection and pre-processing phases. The dataset contains 2800 samples from 14 different categories/classes. These classifications are divided into three groups: (i) vowels, (ii) consonants, and (iii) words. The vowels group consists of the five classes A, E, I, O, and U. The second group consonants are F, G, M, and S, while the last group is made up of words from the Fish, Goat, Meal, Moon, and Snake classes. Each of these groups has classes with an equal amount of samples. The dataset of each group was divided into two subsets: training and testing. In the vowels and words, dataset 300 samples were used as test and 700 samples for training. In the same way, the consonants dataset was divided into two parts, 240 samples for testing and 560 for training. All classes and subjects are represented equally in the training and testing sets. We have obtained ethical approval for the study. The approval confirms that all research was conducted in accordance with relevant guidelines and regulations and includes in the manuscript a statement confirming that informed consent was obtained from all participants and/or their legal guardians. The University of Glasgow’s Research Ethics Committee granted permission for this study (permission numbers: 300200232, 300190109).

### Data pre-processing

The collected data was in the form of RSSI values stored in a single CSV file namely Scikit. The library was used to preprocess data and implement machine learning models. Additionally, CSV files are interpreted using the Python program, i.e., Pandas. The CSV files are then converted into data frames, which are then analysed with SciKit29. In the end, 14 labels were added in the first column of data frames. A total of 9 features were extracted namely, mean, median, mode, standard deviation, variance, minimum, maximum, and high order moments, such as skewness and kurtosis. The final data is fed to different machine learning algorithms, namely Random Forest, K-Nearest neighbor, K-Nearest Neighbors(k-NN), Support Vector Machine (RBF), Logistics Regression, and SVM RBF.

### Evaluation metrics for classification model

The performance of ML models in the classification of three sub-group (vowels, consonants, and words), and combined dataset is evaluated using weighted average accuracy, precision *P*, recall *R*, and F1- score. The equation $F1-Score={2*(P.R)}/{(P+R)}$ is used to calculate the F1 score, one of the most popular classification metrics in the literature,. The F1 Score combines precision and recall, which are calculated using the standard equations, $Precision={\sum (TP)}/{\sum (TP+FP)}$ and $Recall={\sum (TP)}/{\sum (TP+ FN)}$. The equation $Accuracy={\sum (TP+TN)}/{\sum (TP+FP+TN+FN)}$ is used to calculate the Average accuracy, used to evaluate the performance of a machine learning models.

### Classification via machine learning models

For classification, the RSSI information collected in the previous step is fed into Machine learning models. Three different machine learning models are considered for this purpose: Random Forest, k-NN, and SVM(RBF). The high-level signal flow diagram of the proposed Lip- reading recognition system is illustrated in Fig. 3. Our classification framework differentiates different groups of English structures such as vowels, consonants, and Words. The next subsections provide a detailed description of the machine learning models used in this research.

**Table 1:** Selected model parameter configurations.

ML model	Parameter	Setting
Random forest	N estimators	200
	CV	10
	Criterion	gini
	Min sample split	2
	Max feature	Sqrt
	Min sample	1
K-nearest neighbours	N Neigbors	3
	CV	10
	Weights	Uniform
	Leaf size	30
	P	2
	Metric	Minowski
SVM RBF	Gamma	Auto
	Kernel	Rbf
	C	6.7
	Degree	3
	Cache size	200

#### Random forest

A random forest is a cutting-edge machine learning classifier for classifying numeric datasets^27^. In order to fit various decision tree classifiers on various subsamples of the dataset, it implemented a meta-estimator to increase predicted accuracy and manage over-fitting. Table 1 presents the hyper-parameter settings used for the Random-Forest model.

#### K-nearest neighbors (k-NN)

This is a well-known decision rule that is commonly used in pattern classification^28^. In this technique, the ideal choice of the value of k was largely data-dependent; generally, a bigger k decreases the effects of noise but makes the classification boundaries less distinct. The hyper-parameter settings of k-nearest neighbor are shown in Table 1.

#### SVM RBF

RBF kernel SVM used gamma and C parameters^29^, where the gamma parameter defines how far a single training example’s influence reaches, with low values indicating “far” and high values indicating “close.” The C parameter trades off correct training example classification against maximisation of the decision function’s margin. The hyper-parameter settings of SVM RBF are shown in Table 1.

## Results and discussion

**Fig. 7:**
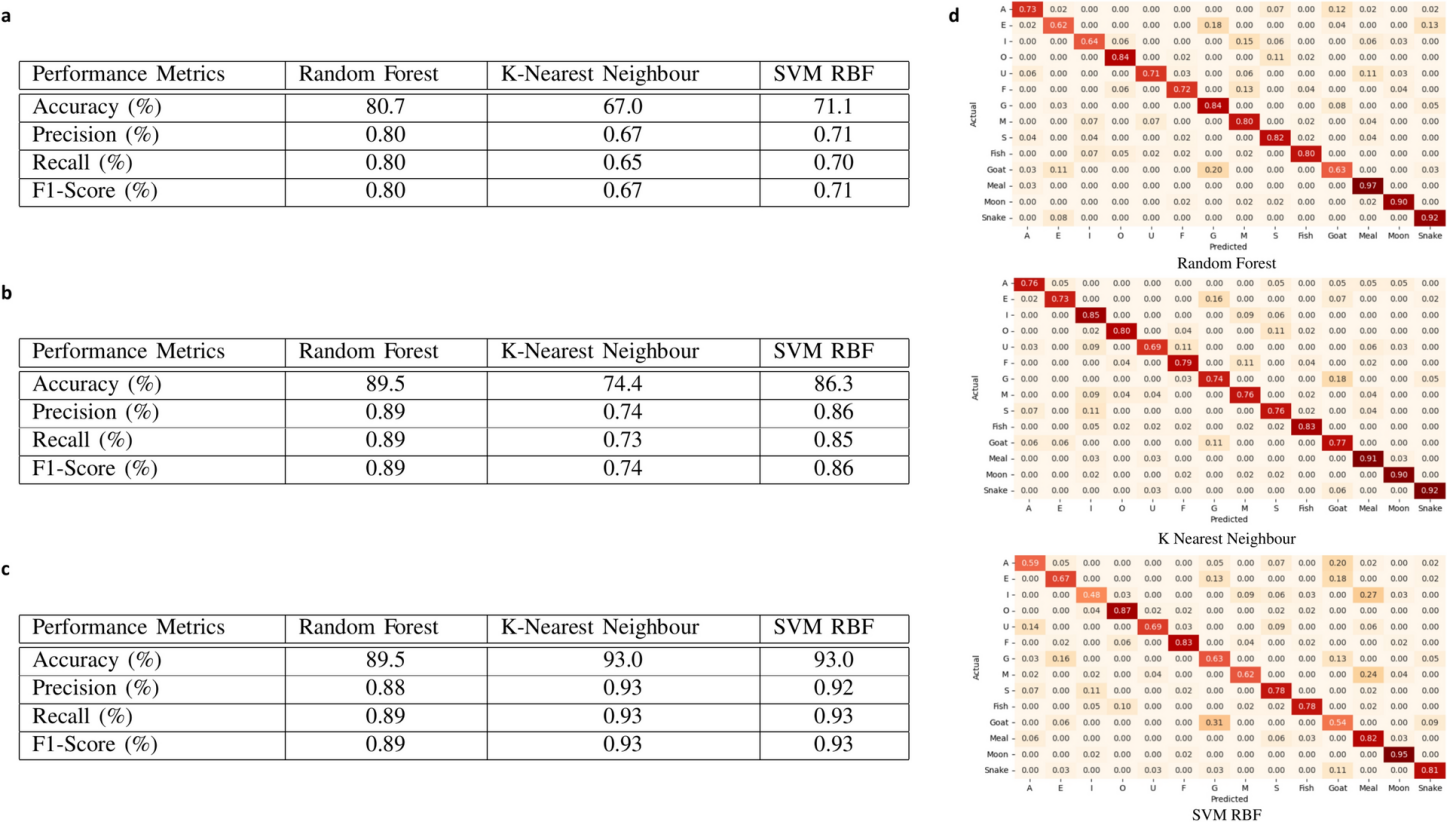
Experiment results of different machine learning models for the classification of Lip-reading. (**a**) Vowels. (**b**) Consonants. (**c**) Words. (**d**) The confusion matrix of the combined result of all fourteen classes.

The experimentation in this work serves two purposes. First, we introduced an RFID-based smart mask for lip-reading recognition. Second, we compared the performance of various existing machine learning models including Randon Forest, k-NN and SVM RBF algorithms. We collected and analyzed different sub-categories of English structure datasets, such as vowels, consonants, and words, from diverse genders to evaluate the performance of RFID-based lip-reading frameworks. As a result, we conducted three distinct experiments on RSSI-captured data to evaluate the models’ performances. Table 1 contains the hyper-parameter settings for all models. All models were fine-tuned on the dataset, using fixed training and testing sets, with the training set containing 80% of the total data and the testing set containing 20%. Figure 7 displays the experimental results of various English language structures in terms of precision, recall, and F1 score. Overall, better results were achieved for the combined and individual groups for all the models.

### Results

For the vowels dataset, we calculated results for diverse subjects, including both females and males. Subjects (S1), (S2), and (S3) achieved high accuracy using the SVM RBF algorithm, with accuracy rates of approximately 97.18%, 83.13%, and 91.96%, respectively, compared to other machine learning algorithms. Subject (S4) also demonstrated high classification accuracy, around 79.91%, using the Random Forest algorithm. In the combined RFID lip-reading vowels dataset of all subjects, both female and male, we achieved a high classification accuracy of 80.0% with precision, recall, and F1-score using the Random Forest algorithm, as shown in Fig. 7a.

Similarly, consonant datasets namely F, G, M, and S were collected by diverse groups of subjects. The Subject (S1) and Subject (S4) have high classification accuracy using Random Forest, k-NN, and SVM RBF algorithm around 97.18% and 97.48% as compared to other machine learning algorithms. Subject (S2) and Subject (S3) have a high accuracy of 86.43% using Random forest and k-NN, than other proposed machine learning algorithms. In the combined RFID consonant datasets, we got high classification accuracy of 89.5% using Random Forest algorithm with high precision, recall, and F1-score as compared to other machine learning algorithms which are shown in Fig. 7b.

In the case of words datasets namely Fish, Goat, Meal, Moon, and Snake were collected by multiple subjects. K-NN algorithm has high classification accuracy of around 89.15% using Subject (S1) and Subject (S3) datasets as compared with other machine learning algorithms. In terms of Subject (S2) and Subject (S3) a high classification accuracy is achieved using the SVM RBF algorithm which is around 95.58% and 97.18% respectively. RFID words combined datasets got 93.0% classification accuracy along with high precision, recall, and F1-score using k-NN and SVM RBF algorithms which are shown in Fig. 7c.

Lastly, the confusion matrix of the combined dataset is shown in Fig. 7d. Three different machine learning models were applied to RSSI information namely Random Forest, k-NN, and SVM RBF. In the case of Random Forest, most of the classes are correctly recognised except “U” because it performed similarly to “F”. Here again, most of the classes are correctly classified using the k-NN algorithm except “I” which was misclassified with “S”. Furthermore, the confusion matrix of SVM RBF mostly classifies all the classes except two classes, “Goat” and “I”. Overall, all three algorithms correctly classified 14 classes but Random Forest outperformed others with 80.0% classification accuracy.

### Discussion

In this study, we propose an RFID-based lip-reading framework that generates signals using an RFID reader, which employs RSSI signals to identify human lip movements across various classes. This RF sensing system can operate as a standalone device or assist hearing aids by detecting lip and mouth movements that often obstruct visual cues in vision-based systems. We collected a diverse dataset from four participants (two males and two females) covering vowel sounds (A, E, I, O, U), consonants (F, G, M, S), and words (Fish, Goat, Meal, Moon, Snake). This dataset was utilized to train several machine learning algorithms. The primary aim of the study was to develop a secure lip-reading system capable of identifying lip movements while wearing a mask in a COVID-19 context using RFID sensing technology and machine learning algorithms. We evaluated three algorithms Random Forest, k-NN, and SVM RBF, using train-test evaluation methods on the RFID dataset, achieving a maximum classification accuracy of 93.0% on the combined dataset. As a proof of concept, this system demonstrates the effectiveness of RFID smart mask technology for lip detection. Future experiments will focus on real-time detection of different words or sentences and exploring various angles of RFID usage. While the RFID-based smart mask improves real-time tracking, data accuracy, and addresses privacy concerns, it faces challenges related to high setup costs and integration. The dataset used to achieve these results has been made publicly available to support further research and development in this field.

Integrating RFID technology into face masks for lip-reading presents a novel solution that addresses privacy concerns and improves performance in low-light conditions, but several practical challenges must be considered for real-world deployment. One significant factor is the cost associated with developing and producing RFID-enabled smart masks, as well as the expenses related to the necessary infrastructure, such as RFID readers and antennas. The cost for RFID infrastructure is very subjective as it varies according to the features and requirements of the application. In general, passive chip-based RFID tags are most cost effective and are preferred due to their low cost and passive features. On the other hand, the RFID readers can vary in performance features and may cost low to high accordingly. Scalability is another critical factor, as the system must be capable of handling a diverse user base with varying needs and speech patterns. RFID has the ability of integration into the IoT infrastructure and by leveraging this integration, it can be scaled to advancing cloud based data processing^30^. Additionally, integrating this technology with existing hearing aids may poses technical challenges, requiring seamless data transmission and synchronization to ensure reliable performance. Comprehensive evaluation and testing are needed to address these challenges, ensuring the system’s feasibility and effectiveness in enhancing communication for individuals with hearing impairments.

## Conclusion and future works

This paper presents a contactless and privacy-preserving lip-reading recognition framework using a passive RFID tag embedded in an everyday wearable mask. Data from the tag is processed by various machine learning models to achieve effective lip-reading recognition. Fourteen different datasets were collected, categorized into three classes: vowels (A, E, I, O, U), consonants (F, G, M, S), and words (Fish, Goat, Meal, Moon, Snake). The experiment involved four participants, two males and two females, aged between 16 and 50 years. The RSSI data from the RFID tag was processed using machine learning models, including Random Forest, k-NN, and SVM RBF. The system demonstrated effective classification of lip movements, achieving a 100% accuracy rate for the datasets. Among the models tested, the Random Forest model performed best, with an overall accuracy of 80.0% across all 14 classes. These findings highlight the potential of using RFID technology combined with machine learning for lip-reading recognition in a contactless and privacy-preserving manner. Future work aims to develop a real-time and intuitive lip-reading system that can recognize a broader range of words and sentences and personalize the system for various end-users, including individuals who are deaf or blind, to enhance its practical application and user accessibility.

## Data Availability

The datasets utilised in the current study are available from the corresponding author upon reasonable request at *qammer.abbasi@glasgow.ac.uk*.

## Footnotes

1 https://voyantic.com/lab/tagformance-pro/tag-designer-suite/.

2 The participants have given their permission for the publication of all the information and images used in this study.
